# Evaluation of Biodentine Pulpotomies in Deciduous Molars with Physiological Root Resorption (Stage 3)

**DOI:** 10.5005/jp-journals-10005-1546

**Published:** 2018-10-01

**Authors:** Hitaf N Nasseh, Balsam El Noueiri, Charles Pilipili, Fouad Ayoub

**Affiliations:** 1 Doctor, Department of Pediatric Dentistry, Lebanese University, Beirut, Lebanon; 2 Associate Professor, Department of Pediatric Dentistry, Lebanese University, Beirut, Lebanon; 3 Professor, Department of Pediatric Dentistry, Catholic University, Louvain, Belgium; 4 Professor, Department of Forensic Odontology and Human Identification, Lebanese University, Beirut, Lebanon

**Keywords:** Biodentine™, Primary molars, Pulpotomy, Pulp canal obliteration, Physiological root resorption.

## Abstract

**Introduction:**

Conservation of primary dentition is essential for maintenance of arch length, esthetic, mastication, speech and prevention of abnormal habits. The commonly supported treatment for retaining carious primary molars with affected pulp is pulpotomy.

**Aim:**

The study aims to evaluate clinically and radiographically the rates of success and efficacy of Biodentine™ as pulpotomy medicament exclusively on primary molars with physiological root resorption.

**Materials and methods:**

A total number of 35 primary molars in stage three of formation were selected to undergo pulpotomy treatment. All teeth were restored with pediatric stainless-steel crowns.

The clinical findings were evaluated at 1, 3, 6 and 12-month intervals and the radiographic follow-ups evaluations were done at 6 and 12 months. The resulting data were tabulated and statistically analyzed using IBM SPSS© for Windows version 20.0 (SPSS, Chicago, IL, USA). Mc Nemar test was conducted to evaluate the differences in results between months 6 and 12.

**Results:**

Periodontal ligament space (PLS) widening and alveolar bone lesion were not seen in any of the 35 cases, 9 teeth (25.7%) manifested pulp canal obliteration (PCO), and none of the cases showed signs of pathologic root resorption. The clinical and radiographic success rates in pulpotomy using Biodentine™ at 6 and 12 months were 100%.

**Conclusion:**

Pulpotomies performed with Biodentine™ were entirely successful. This dressing material appears to be a serious pulpotomy agent in primary molars with root resorption.

**How to cite this article:** Nasseh HN, Noueiri BE, Pilipili C, Ayoub F. Evaluation of Biodentine Pulpotomies in Deciduous Molars with Physiological Root Resorption (Stage 3). Int J Clin Pediatr Dent., 2018;11(5):393-398.

## INTRODUCTION

Conservation of primary dentition is essential for maintenance of arch length, aesthetic, mastication, speech, and prevention of abnormal habits,^1^ hence every effort should be directed to preserve teeth as much as possible. The commonly supported treatment for retaining carious primary molars with affected pulp is pulpotomy.^2^

A unique feature of a temporary tooth is that it goes through three physiological evolutionary stages that influence its reaction to different aggressions. Stage one, also called stage M (stage of maturation process), is the period of root formation. At this stage, the maturing pulp has a strong dentinogenetic and repair potential. Stage two, also called stage S (stage of stability), corresponds to the stability period of the tooth on both the pulp and radicular levels. While stage three, called as well stage R (stage of resorption), consists of the physiological root resorption of the deciduous tooth, up until its loss and the eruption of the underlying successor.^3,4^

Historically, a wide variety of medicaments has been suggested to be used as pulpotomy medicaments. However, formocresol (FC) has been the most popular material for several years. Although there is evidence of clinical and radiographic success,^5^ there is controversy about the toxicity of FC and its carcinogenic potential.^6,7^ Therefore, more biocompatible alternative materials have been proposed.

In 1993, Torabinejad introduced Medical Technical Assistant (MTA), which is composed of tricalcium oxide, tricalcium silicate, tricalcium aluminate, and silicate oxide.^8^ It is biocompatible, has a high sealing ability, ability to form a dentinal bridge and can cause regeneration of cementum and periodontal ligament.^9^ However, it has some associated drawbacks related to mechanical properties, handling characteristics, cost, and composition.^10,11^

To overcome these shortcomings, efforts have led to the development of new calcium silicate based material with adequate biological and mechanical properties called Biodentine™ (Septodont, Saint-Maur-des-Fosses, France) with active bio-silicate technology with the intention of preserving the properties and clinical applications of MTA without its negative characteristics.^12^

Despite all the studies conducted on the effects of different pulp dressing materials in pulpotomy on deciduous teeth, none of them specifies the physiological stage of the roots, especially that stage 3 is a phenomenon that occurs only in the primary dentition.^13^

This study aim to investigate the clinical and radio-graphic outcomes of vital pulpotomy with Biodentine™ in stage 3 deciduous molars exclusively and where at least two of their roots are at half their lengths.

## MATERIALS AND METHODS

This study was conducted in the Department of Pediat-ric Dentistry, at the school of dentistry of the Lebanese University. Before meticulous radiographic and clinical inspection, written consent was obtained from the parent/guardian after explaining the full details of the treatment procedure.

A total of 35 primary molars in 31 healthy children, categorized by number one according to the American Society of Anesthesiologists (ASA1), aged from 8 to 11 years were included in the study; all selected molars were in stage 3 of formation and required pulpotomies. The inclusion criteria are as follows:

- Deep caries
- Mechanically or traumatically exposed primary molars
- No history of spontaneous pain or irreversible pulpitis
- Normal radiographic findings
- Feasible restorative treatment.

The exclusion criteria should include:

- Radiographic evidence of pulp or periradicular pathosis
- History of spontaneous pain
- Excessive hemorrhage encountered during the clinical procedure
- Evidence of calcification in the pulp chamber
- Exposures with purulent discharge or serous exudate
- Presence of fistula
- Pathological mobility.

Initially, the molars were clinically and radiographi-cally evaluated for vital pulpotomy, but the final diagnosis was confirmed intraoperatively through clinical findings. The molars showing profuse bleeding (for more than 5 minutes reluctant for hemostasis by compression) or absence of bleeding were excluded from the study.

Digital periapical radiographs were obtained using Kodak^®^ intraoral machine (model 2100) set at 70 Kv with 0.3 seconds of exposure time for maxillary molars and 0.2 seconds for mandibular molars. The radiographs were taken at:

- *t (-1):* Preoperatively during the first examination consultation,
- *t (0):* After pulpotomy and applied stainless steel crown (SSC),
- *t (1):* At the first radiologic follow-up, after 6 months,
- *t (2):* At the second radiographic follow-up, after 12 months.

One operator performed all the pulpotomy procedures. The clinical success was defined by the absence of spontaneous pain, swelling, sinus tract, and abnormal mobility.

The radiographic success criteria included the normal development of the successor, the presence of normal PLS, the absence of bone lesion and pathologic root resorption.

On clinical and radiographic examinations, if any of these criteria were observed, the treatment was recorded as unsuccessful. Moreover, any radiographic evidence of PCO was not regarded as a failure and physiologic exfoliation of primary molars after six months following a vital pulp therapy was counted as a success.

### Clinical Procedure

After administering local anesthesia with Lidocaine (2%), a quadrant isolation was performed with a rubber dam. All superficial caries were removed with a sterile carbide fissure bur mounted on a high-speed water-cooled hand-piece. Sterilized and disinfected instruments were used. Following the standard of care in pulpotomy treatment, no disinfection of the coronal cavity was performed. Coronal access was gained by joining the pulp horns with a high-speed bur under continuous irrigation.

After attaining pulp chamber exposure, a carbide round bur, mounted on a low-speed handpiece, was used to remove the superficial coronal pulpal tissues while the rest of the coronal pulp amputation was done with a sharp sterile spoon excavator. The chamber was flushed with 5 cc sterile saline and a series of sterile cotton pellets moistened with saline were placed over the pulp stumps under light pressure for 2 to 3 minutes to achieve hemostasis. The cotton pellets were removed, Biodentine™ was used according to the manufacturer’s instructions. The paste was obtained by mixing premeasured unit dose capsules for 30 seconds at 4200 rpm in a titrator and placed over the pulp stumps using an appropriate spatula delivered in the box by the manufacturer.

After 12 minutes Biodentine™ set, a thick mix of zinc oxide eugenol cement intermediate restorative material (IRM) was placed to fill the access cavity for hermetic sealing.

The tooth was restored in the same session with a stainless steel primary molar crown (3M Unitek SP^®^, USA) with a well fitted marginal adaptation and cemented with glass ionomer cement (Ketac-Cem; 3M ESPE, USA).

At the end of the treatment session, the data were recorded on a form for each patient.

All teeth were followed up clinically at 1, 3, 6 and 12 months and radiographically at 6 and 12 months.

In case of loss of the SSC during the follow-up period, the tooth was excluded from the study.

The clinical success was determined by the absence of an abnormal mobility, a swelling, a spontaneous pain or a sinus tract.

The postoperative radiographic success was evaluated by the absence of widened PLS, pathological root resorption or bone lesion.

All clinical and radiographic data were collected.

As the clinical data after 12 months did not show any specified symptom of failure, no statistical analysis was carried out.

Radiographically, 35 cases were studied to see if there is:

- A PLS widening, bone lesion, radicular pulp lumen thinning or pathologic root resorption or abnormal development of the successor.
- A difference in the outcome at the end of the 6thmonth and the 12th one.

**Graph 1:**
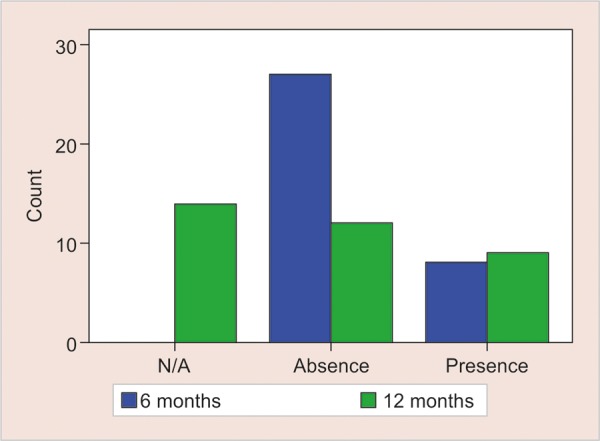
Status of radicular pulp lumen thinning results from radiographic follow- ups at 6 and 12 months

To evaluate the radiographic variables collected in Graph 1 and Table 1, a statistical analysis was performed using IBM SPSS for Windows version 20.0 (SPSS, Chicago, IL, USA). Descriptive statistics and Mc Nemar test was conducted to evaluate the differences of variables at 6 and 12 months. A p < 0.05 was taken to state statistical significance.

## RESULTS

### Clinical Findings

Among the 35 teeth treated with Biodentine™, no tooth revealed abnormal clinical findings at the end of 6 months.

At the 12-month follow-up period, 14 teeth were absent due to normal exfoliation. The 21 remaining molars were free of any symptom of failure.

### Radiographic Findings

PLS widening, bone lesion, radicular pulp lumen thinning, pathologic root resorption and development of successors were evaluated for 35 cases.

### After 6 Months

PLS widening and bone lesion were not seen in any of the 35 cases. The radicular pulp lumen thinning was observed in 22.9 % of the cases (n = 8). No pathological root resorption was noticed in any of the cases and all the successors were progressing normally.

**Table 1:** **Table 1:** Radiographic results at 6 and 12 months

				*6 months*						*12 months*					
*Parameters*		+		–		N/A		+		–		N/A			
Periodontal ligament space widening		0		35 (100%)		–				21 (60%)		14 (40%)		p > 0.05	
Status of bone lesion		0		35 (100%)		-		0		35 (100%)		–		p > 0.05	
Radicular pulp lumen thinning		8 (22.9%)		27 (77.1%)		–		9 (25.7%)		12 (34.3%)		14 (40%)		p > 0.05	
Pathologic root resorption		0		35 (100%)		–		0		35 (100%)		–		p > 0.05	
Normal development of successor		35 (100%)		–		–		21*(60%)14** (40%)						p > 0.05	

*presence of successor; **eruption of successor

+ Positive; - negative; N/A not applicable.

*Note:* N/A define the cases that could not be evaluated due to some physiologic factors such as eruption of successor or physiologic root resorption.

### After 12 Months

No bone lesion was seen in any of the cases. A total of 40% of the deciduous molars (n = 14) were exfoliated and could not be evaluated for PLS widening and PCO. Concerning the persistent 21 teeth, the radicular pulp lumen thinning was observed in nine cases (Graph 1). Otherwise, no PLS widening, bone lesion or pathological root resorption were detected (Table 1).

There was no significant difference (p > 0.05) between 6 months and 12 months in terms of outcomes of the treatment.

## DISCUSSION

The present study was conducted to evaluate the preliminary effects and success rate of Biodentine™ as a pulp-dressing agent during pulpotomy in stage 3 deciduous molars with advanced physiological root resorption. Scarola (2001) defined the root resorption as precocious when this resorption does not exceed 1/3 of the root surface and as advanced when it is exceeding the third.^14^ At this latter stage, the deciduous teeth lose their potential for sensation, healing, and repair, which makes focusing on the characteristics of Biodentine™ easier.

The ideal pulpotomy material must possess certain characteristics. It must be bactericidal, harmless to a pulp and surrounding structures, promote healing of radicular pulp without interfering with the physiologic root resorp-tion and absent of any toxicity.^15-17^

In recent years, the introduction of new bio inductive and regenerative dental materials like Biodentine™ has witnessed new innovations in dentistry.^12^ There is ample evidence for its positive effects in contact with vital pulp tissue.^12,18^ It has the potential to induce apposition of tertiary dentin by stimulating odontoblasts and promotes the early formation of reparative dentin by induction of cell differentiation.^12,18,19^

Biodentine™ has excellent antimicrobial properties because of its high pH (pH = 12), has high biocompat-ibility and bio-activity.^19,20^ Collado-Gonzalez et al. demonstrated that Biodentine™ exhibited better cyto-compatibility and bioactivity than MTA Angelus on stem cells from human exfoliated primary teeth.^21^ This has prompted its use for pulpotomy.

The role of SSC restoration over pulpotomized teeth is to protect the underlying pulp against microleakage.^12,17^ Croll and Killian have recommended SSCs for final restoration of treated molars based on the assumption that there is less leakage in crowned teeth than those restored with amalgam. They recommended SSCs for the restoration of pulpotomized primary molars to minimize the leakage for the long-term success of pulp therapy.^12,22^

After one year of follow-up, none of the patients showed any abnormal clinical findings; the selected teeth were clinically asymptomatic at all examination intervals; no sinus tract, no spontaneous pain, no tooth mobility and no sign of infection was reported.

Despite the low potential of healing of the selected molars, Biodentine™ could ensure stability and pathology-free frames until the physiological exfoliation of the primary molars and the eruption of their successors.

Biodentine™ has a significant potential for pulp healing, excellent sealing property and no cytotoxic effects on pulp cells or PDL.^12^

The comparable success rate for Biodentine™ has been reported in the literature even though none of them was conducted on one specific stage of physiological root status.^16,17,23^

At the 12-month follow-up, Rajasekharan et al. obtained with a smaller sample size (25 cases) 96% of clinical success with Biodentine™ and 100% with ProRoot WMTA, in 29 cases.^17^

For Kusum et al., the overall clinical success rate evaluated for MTA and Biodentine™ over nine months follow-up were 100%.^12^

Cuadros-Fernandez et al. found 97% of clinical success after 12 months of treatment with MTA, whereas the clinical success rate in Biodentine™ group was 100%.^23^

Moreover, by using Biodentine™ as pulp-dressing material, El Meligy obtained 100% of clinical success in all pulpotomized teeth.^20^

Considering the radiographic evaluation at 6 and 12-month follow-up periods, the present results did not show any PLS widening, bone lesion or pathological root resorption. The 100% radiographic success of Biodentine™ is in line with the success rate at 12 months observed, in 2016, by El Meligy (20), while Rajasekharan et al. reported a slightly lower percentage (96%).17

At 12 months, 40% of the molars were physiologically exfoliated. Therefore PLS widening and PCO were not evaluated for these teeth.

Coll et al. defined that a tooth exfoliated greater than six months after vital pulp therapy was always counted as a success in all future time frames.^24^

Out of 35 treated primary molars with Biodentine™, eight teeth(22.9%) showed PCO at the radiographic evaluation at 6 months. One more pulp obliteration case was noticed at the 12-month radiographic evaluation.

A PCO with dentin apposition by the extensive activity of odontoblast-like cells is interpreted as a positive reaction to stimulation and a sign of healing. It is an indicator that the tooth is retaining some degree of pulp vitality and function over time.^12,18^

This percentage of PCO is lower than the one reported by Rajasekharan et al. (2017) who obtained a percentage of 48% for the same follow-up period.^17^ Kusum et al. noticed that PCO was the most common radiographic finding in both MTA (20%) and Biodentine™ (16%).^12^

In our study, as there was no bone lesion or pathological root resorption in all cases at all time-frames, Biodentine™ appeared to be harmless to the normal development of the successor’s teeth.

Smith et al. stated that the permanent successor will only be affected if the radiographic pathology present involves an osseous change.^25^

In 2016, Cuadros et al. obtained a similar result; the use of Biodentine™ lead to 94.9% radiographic success, one molar showed an internal root resorption and a second one exhibited periradicular radiolucency.^23^

Rajasekharan et al. (2017) have reported 94.4% of radiographic success and 5% of the perforated form of internal root resorption with Biodentine™.^17^

The radiographic observation over 9 months, in MTA and Biodentine™on pulpotomized primary molars in a study conducted by Kusum et al. (2015) showed 4% of non-perforated internal resorption in pulpotomized primary teeth cases each in Biodentine™ and MTA groups. Moreover, external root resorption was observed in 8%of the Biodentine™ group and in 4% of the MTA group.^12^

In the present study, clinical and radiographic successes could be attributed to the proper case selection, the availability of patients at scheduled follow-up visits, the high aseptic standards, the correct protocol and the role of the final restoration in preventing microleakage. Moreover, it could be due to the biocompatibility, bioac-tivity and sealing ability of Biodentine™. This material has the ability to maintain a successful marginal integrity due to the formation of hydroxyapatite crystals at the surface which enhances the sealing ability.^26^

De Rossi et al. (2014) declared that the formation of thicker mineralized tissue during pulp repair is in accordance with the quick setting time of Biodentine™.^27^ Jong Ryul Kim et al. demonstrated the potential of Bioden-tine™ for bioactivity by producing an interfacial layer on the root canal dentine in simulated body fluid.^28^

Moreover, Biodentine™ releases a substantial amount of calcium ions during the initial setting time and reduces long-term ion release, thereby producing favorable conditions for pulp repair.^17,21,28^

## CONCLUSION

From the results of the present study, Biodentine™ demonstrated excellent treatment outcomes in pulpotomy of human stage 3 primary molars over 12 months of follow-up. Despite the reduced healing potential of the selected molars, this pulp-dressing material appeared to be a very effective agent in pulpotomy with clinical and radiographic promising results.

At all the examination intervals, no sign of failure was reported. The PCO noticed in nine cases is an indicator that the tooth is retaining some degree of pulp vitality and function over time. Moreover, Biodentine™ offers many advantages (shorter setting time than other materials, enhanced compressive strength, micro-hardness, lower cost) and represents a promising material for use in pulpotomy. Considering the obtained positive result and the characteristics of Biodentine™, this pulp dressing material could be highly recommended for the clinical practice.

It would be interesting to conduct a new study to evaluate the outcomes of Biodentine™ on stage 2 primary molars and to compare the results with those obtained in the present one.
